# Natural Cytotoxicity Receptors: Pattern Recognition and Involvement of Carbohydrates

**DOI:** 10.1100/tsw.2005.22

**Published:** 2005-02-23

**Authors:** Angel Porgador

**Affiliations:** Department of Microbiology and Immunology, Faculty of Health Sciences and Cancer Research Center, Ben-Gurion University of the Negev, Beer-Sheva 84105, Israel

**Keywords:** natural killer, natural cytotoxicity receptor, tumor, virus, heparan sulfate, hemagglutinin, pattern recognition

## Abstract

Natural cytotoxicity receptors (NCRs), expressed by natural killer (NK) cells, trigger NK lysis of tumor and virus-infected cells on interaction with cell-surface ligands of these target cells. We have determined that viral hemagglutinins expressed on the surface of virus-infected cells are involved in the recognition by the NCRs, NKp44 and NKp46. Recognition of tumor cells by the NCRs NKp30 and NKp46 involves heparan sulfate epitopes expressed on the tumor cell membrane. Our studies provide new evidence for the identity of the ligands for NCRs and indicate that a broader definition should be applied to pathological patterns recognized by innate immune receptors. Since nonmicrobial endogenous carbohydrate structures contribute significantly to this recognition, there is an imperative need to develop appropriate tools for the facile sequencing of carbohydrate moieties.

## INTRODUCTION

The hallmark of the innate immune system is the recognition of pathogen-associated molecular patterns (PAMPs) by a limited number of germline-encoded receptors[1]. Innate immune receptors, such as the toll-like receptors (TLRs), have evolved such that they recognize conserved molecular structures (e.g., lipopolysaccharide) produced by microbial pathogens, but not by the host[1,2], yet microbial distinctiveness is not a prerequisite for TLR recognition. For example, TLR-7 recognition of influenza virus ssRNA in the endosomal pathway is probably due to targeting of ssRNA to this pathway (following virus degradation) and not due to recognition of a particular viral RNA motif[3]. Thus, it is possible that innate immune receptors may be triggered by molecular structures that are not exclusive to microbes. Consequently, the definition of PAMPs should probably be extended to cover host-related molecular structures associated with overexpression, stress, and aberrant timing or localization.

Natural killer (NK) cells constitute a highly specialized lymphoid population functionally identified by its potent cytolytic activity against foreign, tumor, and virus-infected cells[4]. Being a fundamental component of the innate immune system, the NK cell recognizes and is triggered by molecular patterns, such as those of transformed or virus-infected cells. The triggering of NK cells is delicately regulated by the balance between opposing signals delivered by inhibitory and activating receptors[5]. Detection of self major histocompatibility complex (MHC) class I molecules expressed on host normal cells by a multitude of NK inhibitory receptors stimulates the NK inhibition signal. In opposition, expression of insufficient amounts of self class I molecules reduces the signaling of the inhibitory receptors. The latter situation has been designated as “missing self” pattern recognition by NK cells; it is frequently a consequence of tumor transformation or of viral infection and it is clearly the state for foreign cells[6].

## NATURAL CYTOTOXICITY RECEPTORS

When the signaling of inhibitory receptors is absent or reduced, the ground is then set for NK-mediated target-cell lysis by the activation receptors[5,7,8,9,10,11]. Major players in this field are the natural cytotoxicity receptors (NCRs) that trigger the NK-cell lysis of various tumor and virus-infected cells through direct engagement of ligands expressed by the target cells. Three novel NK-specific triggering surface molecules — NKp30, NKp44, and NKp46 — have been identified. These molecules belong to a group of receptors collectively termed NCRs. All of them are capable of mediating direct killing of a broad spectrum of tumor and virus-infected cells and are specific for non-MHC ligands[5,12]. The questions, then, are: What is the nature of the tumor or virus-induced ligands recognized by the NCRs and whether these ligands, for each NCR, are identical moieties on all types of target cell or whether they constitute a pattern representing virus-infected or transformed cells?

## VIRAL LIGANDS RECOGNIZED BY NCRS

We have previously found that the NKp44 and NKp46 proteins, but not the NKp30 protein, recognize the hemagglutinin (HA) of the orthomyxo influenza virus and the hemagglutinin-neuraminidase of the paramyxo sendai virus[13,14,15]. The recognition of hemagglutinin and hemagglutinin-neuraminidase by NKp44 and NKp46 is required for the lysis of the virus-infected cells by NK cells[14,15]. This recognition is not limited to the hemagglutinin of a particular strain of influenza virus; in a study of seven different strains of influenza virus expressing different H1- and H3-type HAs, we found that all were recognized by NKp44 and NKp46[16]. We have also shown that NCRs recognized viral proteins with a hemagglutinin function for two additional virus families (A. Porgador, unpublished results). It is known that viral hemagglutinins are sialic acid–binding lectins. We have shown that the binding of the NKp44 and NKp46 NCRs to different viral hemagglutinins requires the sialylation of NKp44 and NKp46 oligosaccharides[14,15]. We have further demonstrated that this recognition is restricted to one of the three glycans found on the second extracellular domain of NKp46[16]. Given that members of at least seven virus families utilize sialic acid as a receptor for virus entry into host cells and that viral hemagglutinins are expressed on the surface of infected cells, it is likely that there is a general strategy for NK cell recognition of a virus-infected cell pattern induced by a substantial subset of viruses.

## TUMOR CELL RECOGNITION BY NCRS

A number of researchers, including our group, have shown that NKp30, NKp44, and NKp46 NCRs recognize cellular ligands expressed on a wide variety of tumor cell lines[7,12,14,15,16]. As is the case for normal cells, human peripheral blood leukocytes were negative when stained with different recombinant NCR-Igs[15]. Primary cancerous, but not normal, tissues were also stained by NCR-Igs (A. Porgador, unpublished results). Since NCRs play an important role in recognizing and killing tumor cells, the elucidation of the nature of the tumor ligands recognized by NCRs is imperative. We have recently shown that heparan sulfate epitopes on membrane-associated heparan sulfate proteoglycans (HSPGs) are recognized by NKp30 and NKp46[17]. We have also shown that tumor cells expressing cell-surface heparanase, CHO cells lacking membrane heparan sulfate, and glypican 1–suppressed pancreatic cancer cells manifested reduced recognition by NKp30 and NKp46 and were lysed to a lesser extent by NK cells[17].

Ligands to NCRs may be expressed primarily as a consequence of cellular stress, activation, viral infection, or tumor transformation[18]. The interaction of NCRs with heparan sulfate epitopes on HSPGs can facilitate the binding of the NCRs to other, hitherto elusive, cellular ligands. For example, the heparan sulfate epitopes serve as coligands for NCRs in a way similar to the heparin/heparan sulfate interaction with growth factors and growth factor receptors or with lipid-binding proteins[19,20]. In this case, the heparan sulfate epitopes recognized by the NCRs could be identical/similar for the three NCRs, while the primary cellular ligands (proteins? or lipids?) could differ for the three NCRs, which would explain the functional observations indicating different ligands for different NCRs[4,12]. Recognition of stromal cells by CD19, a coreceptor of the B-cell receptor, was shown to involve HSPGs expressed by the stromal cells[21]. Alternatively, tumor-modified heparan sulfate can overexpress certain existing epitopes or express separate unique carbohydrate epitopes recognized distinctively by NCRs and rarely expressed on membrane heparan sulfate of normal cells. In this scenario, the heparan sulfate epitopes recognized by the different NCRs could be dissimilar. Yet, the exact sequences and the cross-reactivity of these epitopes remain to be explored. Indeed, heparan sulfate exhibits a considerable number of unique overlapping sequences with peculiar sulfation profiles and these sequences are recognized by specific complementary proteins[22]. If unusual heparan sulfate epitopes on cancer cell membranes lead to better signaling through growth factor receptors, it might be that NK-expressed NCRs have evolved to recognize and penalize the “uncommon” epitope-expressing transformed cells. If so, this recognition can be considered as transformed cell pattern recognition by NK cells.

## CONCLUDING REMARKS

Carbohydrate-recognizing receptors constitute a significant part of the innate immune system and its typical pattern recognition features[23]. NK cells recognize patterns apparent on transformed and virus-infected cells. This recognition involves a multitude of NK inhibitory and activator receptors. We have shown that for pattern recognition by NCRs, carbohydrates on both the NCRs and the target ligands play a vital role. Certain sialic acid–bearing glycans on NCRs are crucial to the physiological recognition between NK cells and virus-infected cells expressing viral sialic acid receptors. Therefore, the definition of PAMPs can indeed be extended to include microbial function (recognition by the hemagglutinins) as patterns recognized by innate immune receptors (e.g., NKp44 and NKp46). While the presence of functional hemagglutinin in virus-infected cells creates an obvious marker of alteration, the molecular mechanisms behind possibly altered recognition of heparan sulfate through tumor transformation are still largely unknown. Heparan sulfate glycosaminoglycans (HS-GAGs) play an essential role in key biological processes, most exploited by the cancer for its survival and progress. Changed amounts of heparan sulfate in tumor cells could result in altered recognition, eventually in combination with other ligand molecules. Structural alteration of heparan sulfate due to biosynthetic reasons or tumor-induced expression of modifying enzymes could also lead directly to altered recognition by NCRs or to altered binding properties for protein ligands, which in combination could give new epitopes for NCRs.

The complex heterogeneity of glycans, the presence of different glycoforms, and the lack of adequate tools to assign sequences to carbohydrate structures restrict studies that attempt to elucidate the contribution of carbohydrates to pattern recognition. Yet, it is clear that glycome exploration is a key challenge in the efforts to elucidate major biological topics such as the receptors and ligands governing the pattern recognition of the innate immune system.

## ACKNOWLEDGMENT

The studies described here were supported by grants from Prostate Cancer Foundation Israel, the Cancer Research Institute (CRI), the Israel Cancer Research Foundation (ICRF), and the Israel Science Foundation (ISF).
